# Mechanistic Insights into NO Releasing by Functionalized Carbon Quantum Dots: A DFT Study

**DOI:** 10.1021/acsomega.5c06567

**Published:** 2025-10-31

**Authors:** Henrique Rodrigues Souza-Silva, Orisson Ponce Gomes, João Pedro Dionizio, Didier Bégué, Paulo Noronha Lisboa-Filho, Augusto Batagin-Neto

**Affiliations:** † São Paulo State University (UNESP), Graduate Program in Materials Science and Technology (POSMAT), Bauru, SP 17033-360, Brazil; ‡ Université de Pau et des Pays de l’Adour, E2S UPPA, CNRS, Institut des Sciences Analytiques et Physico-chimie pour l’environnement et les matériaux (IPREM), 64000 Pau, France; § São Paulo State University (UNESP), School of Sciences, Bauru, SP 17033-360, Brazil; ∥ São Paulo State University (UNESP), Institute of Sciences and Engineering, Itapeva, SP 18409-010, Brazil

## Abstract

The development of carbon quantum dots (CQDs) for photoresponsive nitric oxide (NO) delivery is a rapidly advancing field, with experimental reports demonstrating promising release under visible light irradiation. However, a mechanistic understanding of the photolytic process, the explicit role of CQD functionalization, and the influence of key physiological variables such as pH has been lacking, hindering rational design. This study aims to unravel the atomistic details of the NO release mechanism in functionalized CQD systems. Using density functional theory (DFT) and time-dependent DFT (TD-DFT) calculations, we systematically investigated a model system (CQD_CA_^CYS+TPP^···NO) to probe ground-state reactivity, protonation effects, and excited-state properties. Our results reveal that cysteine deprotonation is a critical effect for S–NO bond formation. TD-DFT calculations evidence a low-energy SNO-localized excited state with predominant *n* → π* character, which drives direct photoinduced electron transfer from sulfur to nitrogen, weakening the S–NO bond and rationalizing the experimental photodynamic response. While very acidic conditions can destabilize the system, it remains stable under physiological pH. These findings provide a mechanistic framework that clarifies the synergistic roles of the CQD, cysteine, TPP, and NO moieties on the nanocomposite, offering foundational principles for engineering next-generation CQD-based platforms with enhanced stability, controlled release efficiency, and reduced off-target effects for biomedical applications.

## Graphical abstract

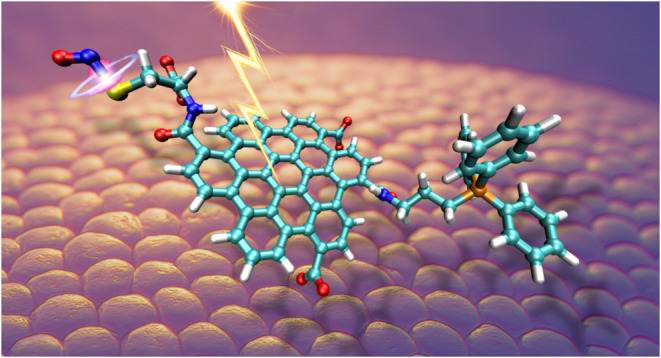

## 1 Introduction

The use of nanomaterials has emerged as a highly innovative strategy for the controlled release and transport of therapeutic agents. Their ability to interact with specific cells and tissues at the molecular level allows for efficient and targeted delivery while minimizing systemic toxicity. The convergence of biomedicine, drug administration, and nanotechnology has thus opened promising avenues for the treatment of pathologies that require precision therapy and controlled intracellular responses.^1,2^

Among these nanomaterials, carbon quantum dots (CQD) have attracted increasing attention due to their unique combination of physicochemical and biological properties. CQDs exhibit remarkable photostability, high quantum yield, tunable surface chemistry, and intrinsic water solubility, which enable their application in areas ranging from chemical sensing and bioimaging to optoelectronics and drug delivery.^3^ Their small size and high dispersibility in aqueous environments not only facilitate cellular uptake but also enhance their ability to act as stable nanocarriers for therapeutic molecules. Importantly, functionalization strategies have been successfully applied to tailor the surface of CQDs for improved biocompatibility, selectivity, and efficiency in drug delivery systems.^4^

Some studies have shown that modified CQDs can be engineered to induce apoptosis in cancer cells by exploiting their surface chemistry and capacity for controlled release.^5^ In particular, Xu et al. demonstrated that CQDs functionalized with S-nitrosothiols (R–SNOs) and triphenylphosphonium (TPP) groups are capable of targeting mitochondria and triggering cell death pathways.^6^ In these systems, R–SNO moieties act as nitric oxide (NO) sources and TPP facilitates the selective accumulation of molecules and nanoparticles within the mitochondria, as its positive charge on the phosphorus atom is retained even when conjugated to other groups.^7^

As a matter of fact, nitric oxide itself can play a dual role in biological systems. At moderate concentrations, NO can stimulate mitochondrial biogenesis and contribute to cell signaling, whereas excessive levels are toxic and promote apoptosis.^8^ Some studies have reported that light-irradiated CQDs can promote rapid and significant NO release, effectively inducing mitochondrial apoptosis. Such effects have been demonstrated in different systems, including CQDs functionalized with *N*-diazeniumdiolates^5^ and mitochondrial-targeted photoresponsive systems.^9^

Despite the promising biotechnological applications of these systems, there is still an absence of in-depth molecular-level analyses that explore how structural, environmental, and chemical parameters can influence the efficiency and dynamics of NO-releasing processes in such nanocomposites. The role of pH is especially critical, as it directly influences the protonation–deprotonation equilibria of functional groups typically present in these systems.^10^ Understanding these effects is essential for designing efficient nanocarrier systems for different physiological microenvironments.

In this context, theoretical studies can define a powerful framework to complement experimental findings and unravel the fundamental mechanisms underlying photoinduced NO releasing. As a matter of fact, a number of computational works have successfully employed density functional theory (DFT)-based calculations to investigate nanomaterials for drug delivery, adsorption phenomena, and NO-releasing platforms.^11–13^ However, to date, there has been a lack of systematic studies focusing on CQD + TPP + SNO systems.

Here, we bridge this gap by performing a comprehensive computational study of a functionalized CQD model system (CQD_CA_^CYS+TPP^), in which NO is anchored at the deprotonated sulfur atom of the cysteine residue. Using DFT and time-dependent DFT (TD-DFT), we evaluate the electronic structure, excited states, stability, and local reactivity of the nanocomposite, with special emphasis on the role of local reactivity, excited-state charge-transfer processes, and pH-induced changes. Our results reveal that system deprotonation is crucial for obtaining an effective S–NO coupling. TD-DFT analysis identifies a key *n* → π* transition around 550 nm that drives a photoinduced electron transfer from sulfur to nitrogen, which facilitates the NO releasing. Our data also shows that, while the stability of the complex is compromised in highly acidic environments, it remains robust under physiological conditions. This mechanistic understanding provides a foundational framework for the rational design of advanced CQD-based platforms.

## 2 Materials and Methods

Figure 1 illustrates the structures evaluated in this report: (i) cysteine amino acid (CYS), which plays a role in NO binding and CQD functionalization; (ii) modified TPP, known for its mitochondrial targeting properties; (iii) nonfunctionalized hexagonal armchair-edged CQD (CQD); (iv) CQD functionalized with four carboxylic groups (CQD_CA_); (v) CQD_CA_ functionalized with CYS and TPP (CQD_CA_^CYS+TPP^); and (vi) CQD_CA_^CYS+TPP^ with trapped NO at (deprotonated) CYS sulfur atom (CQD_CA_^CYS+TPP^···NO).

**Figure 1:**
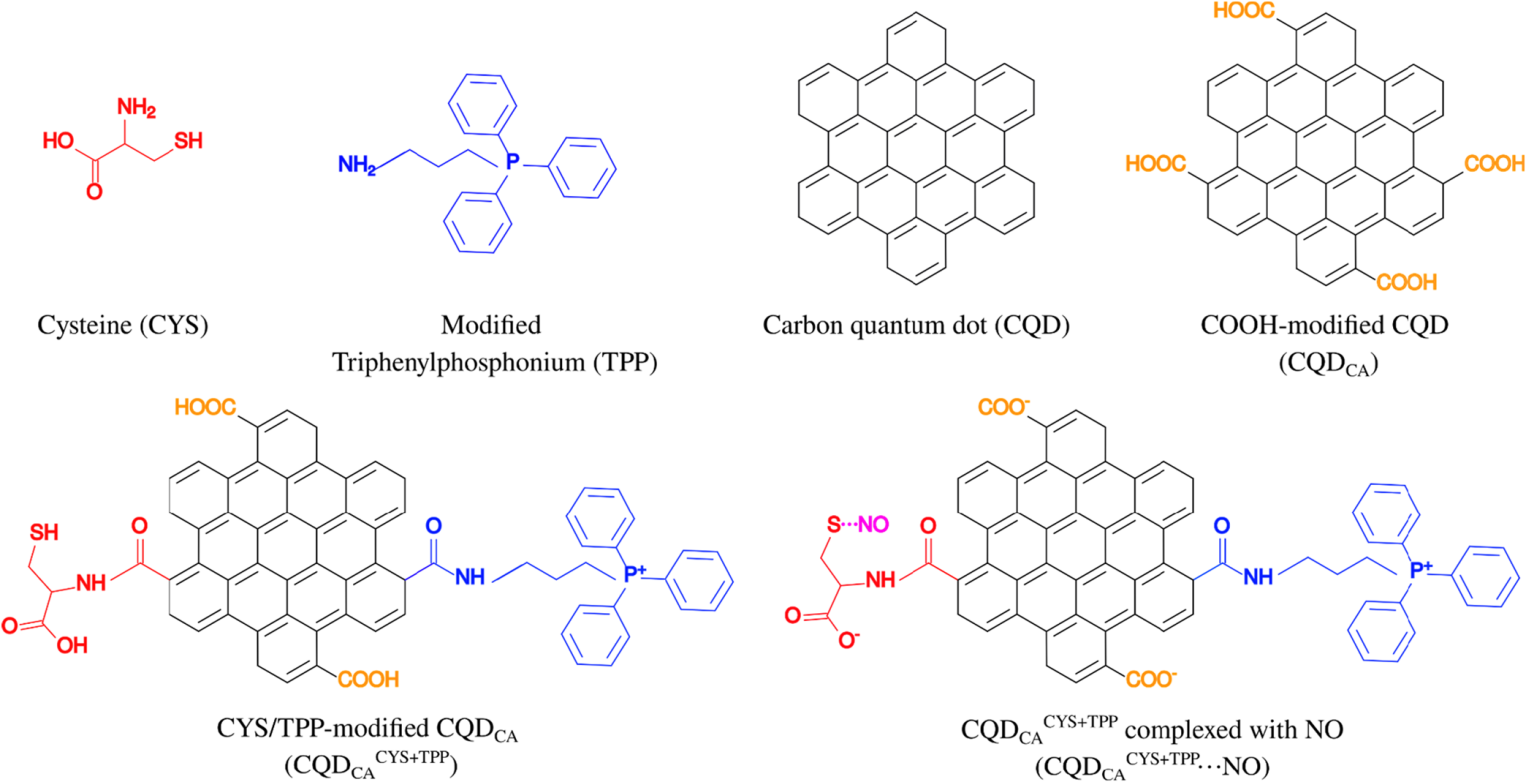
Basic components of the CQD-based NO-carrying system: CYS; TPP; nonfunctionalized CQD; CQD_CA_; CQD_CA_^CYS+TPP^; and CQD_CA_^CYS+TPP^···NO.

CQD_CA_^CYS+TPP^···NO defines the primary model system of this study. To elucidate the specific function of each constituent, supplementary models were constructed and analyzed: (i) isolated cysteine (CYS), (ii) modified TPP molecules, and (iii) modified and unmodified carbon quantum dots (CQD and CQD_CA_). Within the integrated system, each block plays a distinct role: CYS residue provides the anchoring site for NO, critical for the system’s bioactivity;^14^ TPP^+^ groups enables efficient NO delivery by mediating electrostatic interactions with the target mitochondrial membranes,^15^ CQD_CA_ act as a substrate.

Hexagonal structures containing 54 carbon atoms were employed to model the CQD systems. This choice is supported by previous work from our group ,^16^ which demonstrated high edge reactivity toward electrophilic attack in these systems (compatible with COOH functionalization). The COOH functionalization was conducted by successively inserting four carboxylic groups onto the CQD structures, leading to the CQD_CA_ systems. Comparative analysis shows that CQDs with armchair edge terminations outperform the zigzag ones, providing a clear rationale for their selection in this study (see Figures 2 and S1).

**Figure 2:**
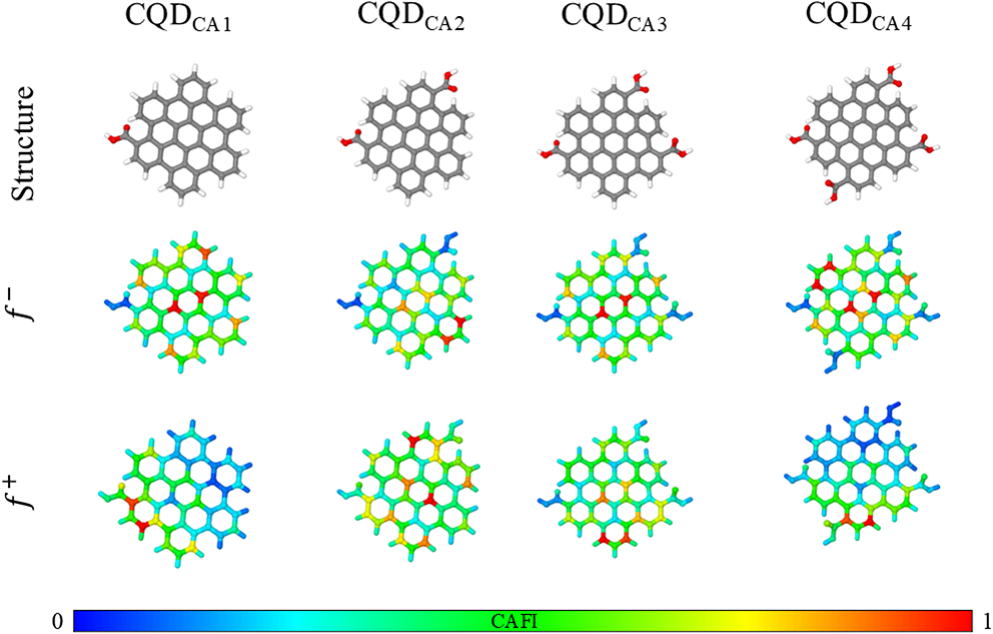
Local reactivity of CQD_CAn_ systems (for *n* = 1 to 4). Red and blue sites represent reactive and inert regions in relation to electrophiles (*f* ^*–*^) and nucleophiles (*f* ^*+*^), respectively.

Following carboxylation, two diametrically opposite carboxylic acid groups were functionalized with CYS and modified TPP, respectively. The final CQD_CA_^CYS+TPP^···NO structure was designed by complexing one NO molecule on the deprotonated sulfur or CYS (via the S···N bond). To simulate different pH conditions, we considered the progressive deprotonation of the carboxylic and thiol groups in accordance with their known p*K*_a_ values^17^ which suggest that these groups are largely deprotonated at or above physiological pH. The final system used in the simulations corresponds to the fully deprotonated form, consistent with the experimental synthesis carried out at pH 7.4.^6^ After deprotonation, NO was added to the sulfur atom of the cysteine residue, forming the S–NO bond.

All of the structures were designed with the aid of GaussView^18^ software. The geometries were optimized at the ground state within the framework of the density functional theory (DFT), employing the hybrid exchange-correlation functional, B3LYP,^19–22^ along with the 6–31G­(d,p) polarized basis set on all of the atoms, with the aid of Gaussian 16 computational package.^23^ In all of the structures, the presence of water was simulated via the polarizable continuum model (PCM) to ensure that the calculated properties and reactivities are representative at physiological conditions. PCM was used as implemented in Gaussian 16, considering the default settings for water (ε = 78.3553, and solvation cavity defined via standard United Atom Topological Model, based on UFF force field radii).^24^

The local reactivities of the components were evaluated via condensed-to-atom Fukui indices (CAFI), in a DFT/B3LYP/PCM/6–31G­(d,p) approach, considering the optimized geometries. Such descriptors allow us to identify how the electron populations around each atom change when the total number of electrons in the system is altered. Three CAFIs can then be defined, representing the local reactivities of the molecule regarding: (i) external nucleophilic agents (*f* ^*+*^), (ii) external electrophilic agents (*f* ^*–*^), and (iii) free radicals (*f* ^0^). In the framework of the conceptual DFT, CAFIs are estimated through finite differences of the atom electronic population, considering the molecule **M** at its neutral (**M**, with *N*_0_ electrons), anionic (**M** ^–^, with *N*_0_ *+ 1* electrons), and cationic (**M** ^**+**^, with *N*_0_ *–* 1 electrons) configurations (see refs 25,26 for details)

1 $${f_{k}}^{+}=p_{k}\left(N_{0}+1\right)-p_{k}\left(N_{0}\right)$$

2 $${f_{k}}^{-}=p_{k}\left(N_{0}\right)-p_{k}\left(N_{0}-1\right)$$

3 $${f_{k}}^{0}=1/2\left[p_{k}\left(N_{0}+1\right)-p_{k}\left(N_{0}-1\right)\right]$$

where, *p*_*k*_ *(N*_0_ *+ 1)*, *p*_*k*_ *(N*_0_ *)*, and *p*_*k*_(*N*_0_ *–* 1) represent the electron populations at the *k*th atom in **M**, **M** ^**‑**^, and **M** ^**+**^, respectively. The atomic populations were calculated using the Hirshfeld charge partitioning method to avoid negative CAFI values.^27,28^ Complementary analysis of CAFI were conducted for CQD_CA_^CYS+TPP^···NO considering distinct partitioning charges schemes (Mulliken and electrostatic potential derived charges—ESP) to assess the robustness and consistency of our results.

The calculation of molecular electrostatic potential (MEP) was also performed for both protonated and deprotonated molecules CQD_CA_^CYS+TPP^, as well as for CQD_CA_^CYS+TPP^···NO complexes, considering the CHELP method (charges from electrostatic potentials using a grid-based fitting approach),^29^ with the aid of Gaussian 16 computational package. Additional calculations considering dimethyl sulfoxide (DMSO) (ε = 46.826) were conducted for the CQD_CA_^CYS+TPP^···NO system to evaluate solvent effects on the local reactivity (CAFI and MEP). The local reactivity descriptors were estimated at the ground state.

Theoretical optical absorption spectra calculations and evaluation of excited states were conducted for CQD_CA_^CYS+TPP^···NO system via a time-dependent DFT (TD-DFT) approach considering two distinct functionals: traditional B3LYP and long-range corrected exchange-correlation functional, ωB97X-D.^30,31^ The ωB97X-D functional combines long-range correction (LC) and empirical dispersion correction (D), which are supposed to mitigate the underestimation of long-range exchange interactions expected for B3LYP, and improve the description of van der Waals and other noncovalent forces. The inclusion of these features is particularly relevant for systems involving adsorptive interactions and excited states with possible charge-transfer features, as they demand an accurate treatment of spatially separated frontier orbitals and an appropriate description of weak intermolecular forces.^32^ By employing both B3LYP and ωB97X-D, this study contrasts a widely used standard hybrid functional with one explicitly designed to handle long-range exchange and dispersion effects, providing a more comprehensive theoretical framework for the analysis.^33,34^ The 10 lowest singlet excited states were considered for both TD-DFT approaches. Varied postprocessing analyses of DFT and TD-DFT results were conducted with the aid of Multiwfn toolbox^35–37^ .

## 3 Results and Discussion

The functionalization of the CQD with carboxylic groups was performed step by step, in which each chemical modification was followed by geometry optimizations. The analysis of *f* ^*–*^ descriptors guided the incorporation of the carboxylic groups (electrophilic addition) on CQDs. Figure 2 presents the resulting structures, highlighting how the chemical changes impact the local reactivity of the material. In general, sites in red and blue represent regions of high and low reactivity, while other colors indicate sites with intermediate reactivity following an RGB scale. *f* ^*–*^ indicates regions with greater affinity with electrophilic agents.

The CAFI analysis reveals a natural distribution of four diametrically opposite carboxylic groups across the CQD structure. Based on these results, it was decided to functionalize the most distant carboxylic groups with CYS and TPP residues, leveraging their specific characteristics, as described by Xu and co-workers.^6^

In fact, positioning TPP and CYS on opposite sides of the molecule is a strategic choice to minimize the potential electrostatic interference that could arise if these groups were in close proximity. TPP, being a positively charged molecule, has a natural affinity for environments with a negative electric potential.^15^ By distancing it from other charged or polar groups, its interaction with mitochondria is maximized, optimizing delivery of TPP to the intended target. Additionally, the CYS residue must be strategically positioned to ensure that its NO-releasing capability is not impeded by steric or electronic interactions with neighboring functional groups.^38^

Figure 3 shows how the frontier molecular orbitals (FMOs: highest occupied molecular orbital (HOMO) and lowest unoccupied molecular orbital (LUMO)) and the electronic gaps (*E*_gap_) of the systems are modified from unmodified CQD to the CQD_CA_^CYS+TPP^···NO complex, including intermediate species.

**Figure 3:**
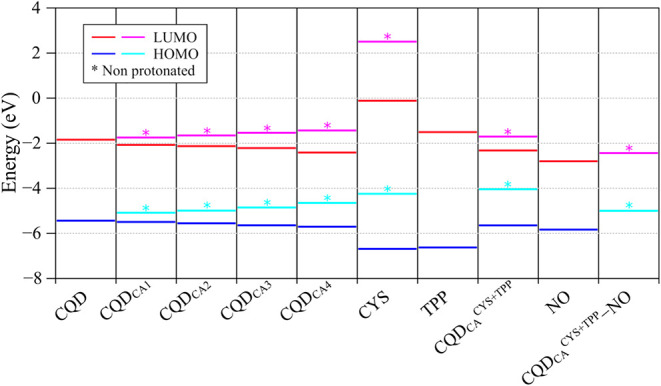
Frontier molecular orbitals of the CQD_CA_^CYS+TPP^ system and its chemical components/modifications.

Note that the addition of COOH groups on CQD leads to a slight reduction of *E*_HOMO_ and *E*_LUMO_, with an opposite effect after deprotonation (CA*_n_* *). It is also interesting to note that the HOMO and LUMO of CYS and TPP are not so close to the CQD_CAn_ systems, so that the frontier levels of CDQ_CA_^CYS+TPP^ are dominated by CQD_CAn_ systems (which shows higher HOMO and lower LUMO than CYS/TPP). This scenario is modified after CYS deprotonation (CYS*), so that CDQ_CA_^CYS+TPP*^ now exhibits higher HOMO levels, dominating the HOMO of the structure and enhancing the local reactivity on this site (see Figure 5). Note that the frontier levels of the CQD_CA_^CYS+TPP^···NO adsorbed system can be considered a combination of HOMO and LUMO of CDQ_CA_^CYS+TPP*^ and NO.

Figure 4 illustrates the density of states (total, TDOS; and partial, PDOS) projected on distinct components of the CQD_CA_^CYS+TPP^···NO complex. In particular, PDOS allows for the identification of each fragment’s contribution to the TDOS, allowing for a detailed understanding of the electronic structure. The dashed lines indicate the energy levels of the frontier orbitals, i.e., the highest occupied and lowest unoccupied molecular orbitals (HOMO and LUMO) at −6.8 and −1.0 eV, respectively.

**Figure 4:**
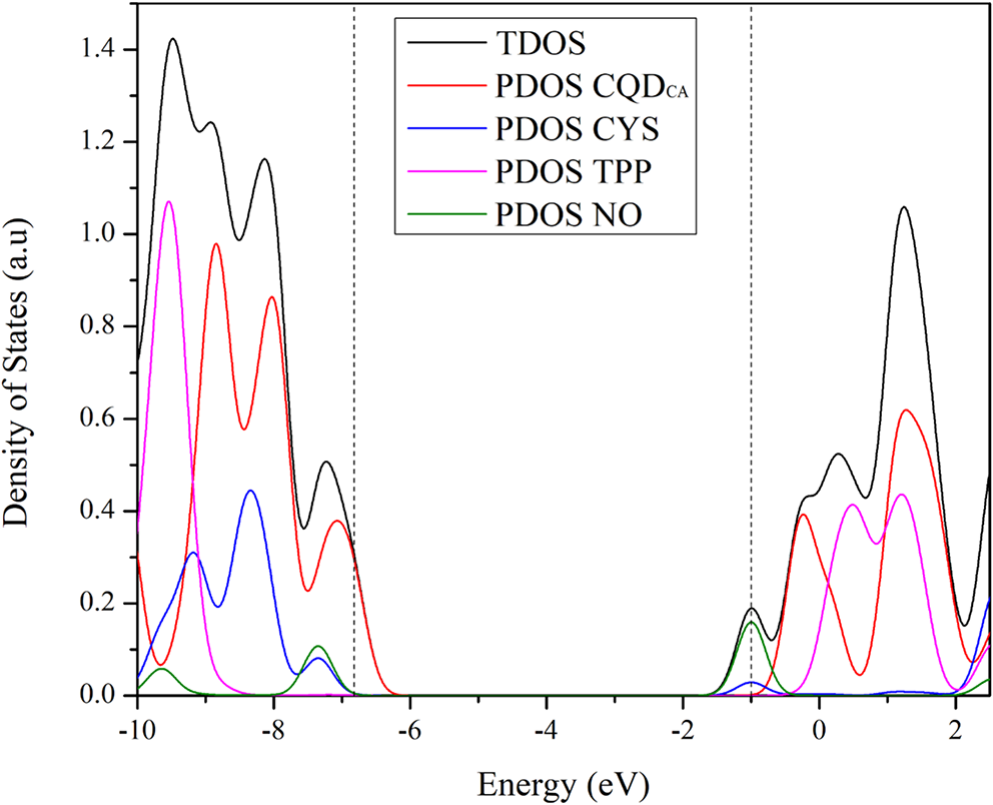
Total and partial density of states projected on each component of CQD_CA_^CYS+TPP^···NO system.

The PDOS clearly reveals a marked separation between HOMO and LUMO in terms of their spatial distribution. The HOMO is predominantly composed of electronic states centered on the CQD_CA_ fragment, with minor contributions from the CYS and NO groups. In contrast, the LUMO is mainly associated with the NO moiety along with a smaller contribution from the CYS fragment. Interestingly, the TPP unit contributes negligibly to the frontier orbitals, which suggests a secondary role in the optoelectronic properties of the system despite its structural presence. Such findings indicate that the CQD_CA_ can act as the primary electron donor in the system, while the CYS + NO acts as the electron acceptors in the ground state.

To better investigate the coupling of nitric oxide (NO) with the CYS residue, CAFI indices were evaluated for CQD_CA_^CYS+TPP^-based systems. The objective was to analyze the local reactivity on these structures and evaluate how it changes due to its protonation state, mainly the reactivity over the CYS anchoring sulfur atom. Figure 5 presents the Fukui indices and MEP for CQD_CA_^CYS+TPP^, its deprotonated structure CQD_CA_^CYS+TPP*^, and deprotonated system with NO trapped in CYS (CQD_CA_^CYS+TPP^···NO). As described by Orth and collaborators,^17^ cysteine-based systems anchored on graphene-like oxides can present an effective deprotonation at slightly alkaline pHs (above 6.59), suggesting that CQD_CA_^CYS+TPP^ systems reported by Xu and co-workers (synthesized at ∼7.4) are supposed to present deprotonated carboxylic groups and sulfur atoms, as illustrated in CQD_CA_^CYS+TPP*^. MEP maps show the distribution of electrostatic potential over the molecule surface (projected on the electron density isosurface). It helps identify regions where the molecule is supposed to interact electrostatically with different chemical species. A red-green-blue (RGB) color scale is used, where red and blue indicate regions of high negative (less positive) and positive (or less negative) electrostatic potentials, respectively. The other colors represent intermediate potentials. Similar results are obtained for the CQD_CA_^CYS+TPP^···NO system considering distinct solvents (water and DMSO), partition charge scheme (Hirshfeld, Mulliken, and electrostatic potential derived—ESP), as well as effective grids for potential estimation (grid size and point densities) (see Supporting Information for details).

**Figure 5:**
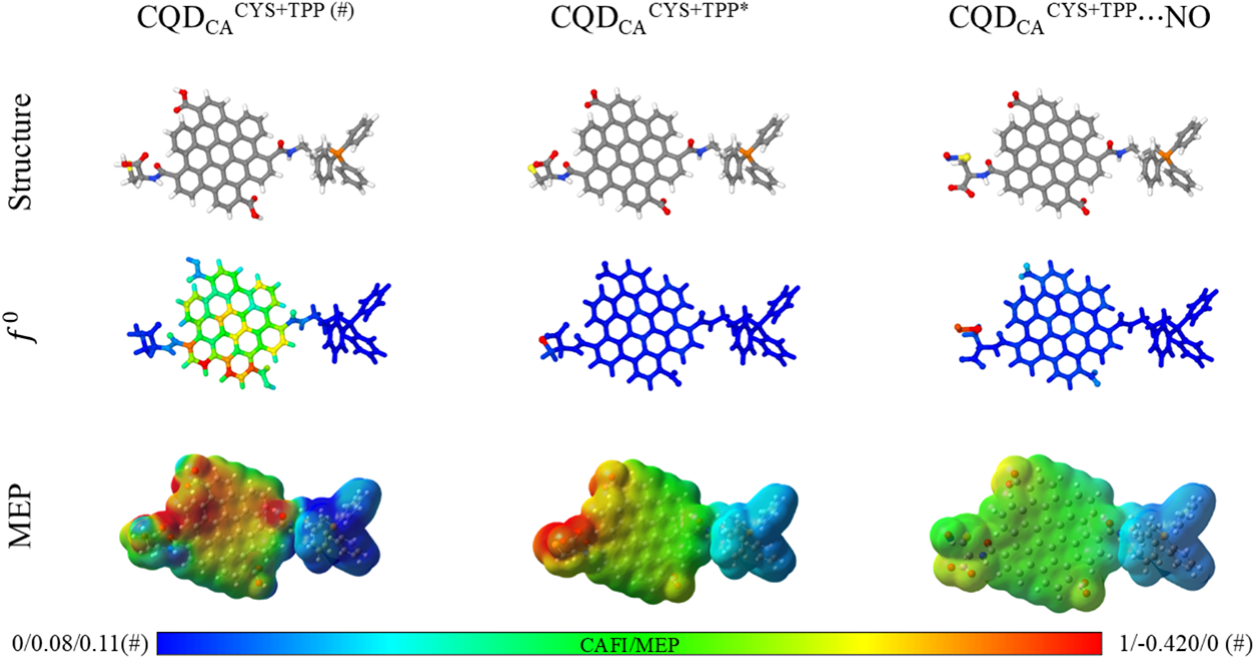
Local reactivity of protonated (#) and deprotonated (*) CQD_CA_^CYS+TPP^ systems and the CQD_CA_^CYS+TPP^···NO complex. For CAFI (MEP), red and blue sites represent reactive (positive potential) and inert (negative potential) regions in relation to radicals, respectively. Different scales were used for the protonated system (#) to better highlight MEP variations.

Note that the sulfur atom does not exhibit significant reactivity in CQD_CA_^CYS+TPP^, suggesting a low probability of forming CYS–NO bonds. Additionally, the reactivity centered over CQD may favor the formation of undesired bonds with other radicals. On the other hand, the deprotonated system exhibits high reactivity at the sulfur atom (compatible with CYS*’s HOMO domination in CQD_CA_^CYS+TPP*^ system—see Figure 3), suggesting a high probability of NO capture. Furthermore, the rest of the structure remains largely inert to radicals, preventing the formation of undesired bonds that could compromise the molecule’s functional mechanisms.

MEP analysis indicates that the TPP consistently retains its positive polarization across all considered scenarios. This characteristic is particularly notable in the case of CQD_CA_^CYS+TPP^···NO, highlighting the crucial role of TPP in effectively targeting mitochondria.

Based on the obtained results, the final system CQD_CA_^CYS+TPP^···NO was modeled, where NO is trapped by deprotonated CYS. It was observed that aside from this bond, the system remains otherwise inert.

To interpret the electronic transitions that could be involved in the photoactivated NO releasing (photoexcitation around 550 nm^6^), TD-DFT-based calculations were conducted for the CQD_CA_^CYS+TPP^···NO model system. Figure 6 illustrates the theoretical absorption spectra of this system estimated in a TD-DFT/ωB97X-D/6–31G­(d,p) approach as well as the most relevant transitions associated with the main peak and those around 580 nm (first excited state—S_1_). Table 1 presents information regarding the first 10 electronic transitions, including the vertical excitation energies, the corresponding peak positions (λ_max_), oscillator strengths (*f*), and identification of the major contributing electronic transitions (with their squared coefficients, *c* ^2^) (see Supporting Information for B3LYP-based results).

**Figure 6:**
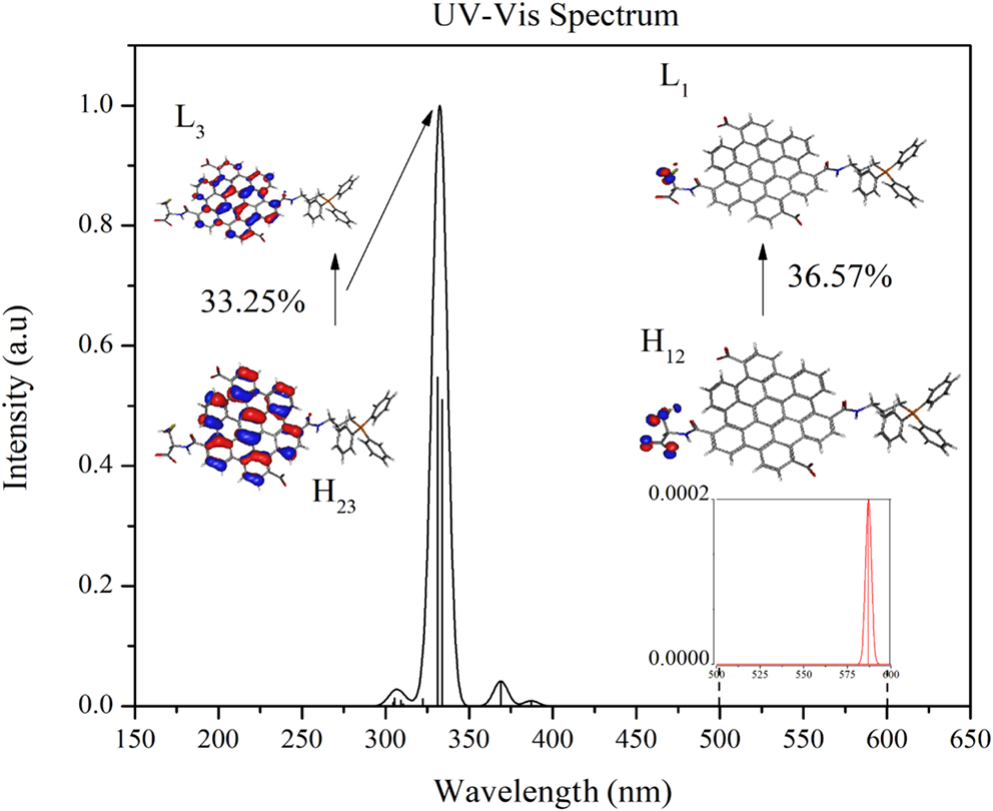
UV–vis spectrum of the CQD_CA_^CYS+TPP^···NO systems. Representation of the orbitals mainly involved in the optical transitions around 331 nm (main peak) and 580 nm (close to experimentally reported NO-releasing photoexcitation).

**Table 1:** First 10 Excited States and Electronic Transitions for CQD_CA_^CYS+TPP^···NO

excited state	energy (eV)	λ_max_ (nm)	*f*	transitions	*c* ^2^
1	2.1120	587.03	0.0002	H_5_ → L_1_	0.0121
				H_11_ → L_1_	0.0691
				H_12_ → L_1_	0.3657
				H_13_ → L_1_	0.0332
2	3.2027	387.12	0.0458	H_22_ → L_17_	0.0107
				H_23_ → L_2_	0.1098
				H_23_ → L_3_	0.0583
				H_24_ → L_2_	0.1207
				H_24_ → L_3_	0.1504
3	3.3607	368.93	0.2141	H_22_ → L_4_	0.0265
				H_23_ → L_2_	0.0588
				H_23_ → L_3_	0.0592
				H_24_ → L_2_	0.1959
				H_24_ → L_3_	0.1152
4	3.7136	333.86	2.6268	H_23_ → L_2_	0.2890
				H_24_ → L_3_	0.1786
5	3.7455	331.03	2.8170	H_23_ → L_3_	0.3325
				H_24_ → L_2_	0.1292
6	3.8468	322.30	0.0610	H_22_ → L_3_	0.0995
				H_22_ → L_9_	0.0154
				H_24_ → L_4_	0.3145
7	3.9937	310.45	0.0171	H_1_ → L_1_	0.1312
				H_2_ → L_1_	0.0125
				H_4_ → L_1_	0.0212
				H_5_ → L_1_	0.0999
				H_6_ → L_1_	0.0655
				H_12_ → L_15_	0.0503
				H_15_ → L_1_	0.0336
				H_17_ → L_1_	0.0257
8	4.0113	309.08	0.0493	H_19_ → L_2_	0.0178
				H_22_ → L_2_	0.2764
				H_22_ → L_3_	0.0402
				H_23_ → L_4_	0.0681
				H_23_ → L_16_	0.0103
				H_24_ → L_17_	0.0105
9	4.0593	305.44	0.0676	H_19_ → L_2_	0.0199
				H_21_ → L_2_	0.0142
				H_22_ → L_3_	0.0713
				H_22_ → L_4_	0.1207
				H_23_ → L_16_	0.0955
				H_24_ → L_17_	0.0309
				H_24_ → L_16_	0.0115
10	4.0721	304.47	0.0294	H_7_ → L_2_	0.0207
				H_10_ → L_3_	0.0147
				H_22_ → L_3_	0.0796
				H_23_ → L_4_	0.2113
				H_24_ → L_4_	0.0260
				H_24_ → L_17_	0.0200

A comparison between the B3LYP and ωB97X-D-based calculations (Tables 1 and S1) reveals that several low-energy transitions with negligible oscillator strengths predicted by B3LYP are absent in the long-range corrected ωB97X-D calculations. These transitions correspond to artificial “dark states” commonly introduced by hybrid functionals.^33,34^ It is worth noting, however, that B3LYP more accurately reproduces the position of the S_0_–S_1_ absorption peak, while ωB97X-D provides consistent state ordering by eliminating spurious excitations. Importantly, ωB97X-D confirms the S_0_–S_1_ transition as the lowest accessible excited state, which is experimentally observed and is directly responsible for the NO-releasing mechanism. This excited state arises from a combination of orbital transitions (dominated by H_12_ → L_1_), and their relatively high λ_max_ values can be associated with the extended π-conjugation of the CQD scaffold with its functional groups. The low oscillator strengths associated with this transition suggests a limited optical accessibility and indicate a *n* → π* character^39^ (see Figure S7). It is in line with experimental observations^6^ and is consistent with the behavior noticed for some type II photoinitiators.^40^

Note that the first excited state of the adsorbed system is centered on the CYS···NO region (inset of Figure 6), suggesting that the experimentally observed NO releasing after photoexcitation (for λ_exc_ ∼ 550 nm) involves electronic transfers in this region. The main absorption peak, observed around 330 nm, involves CQD_CA_-centered electronic transitions; however, it is not accessed by experimental photoexcitation during NO-releasing assay.^6^ It is interesting to note the absence of H-L (dominated) transitions, which reduced H-L superposition, which was identified in Figure 4. Note that both the excited states presented in Figure 6 (S_1_ ∼ 580 nm and S_5_ ∼ 331 nm) are dominated by electronic transitions from deep occupied states (H_12_ and H_23_, respectively) to unoccupied levels close to the LUMO (L_1_ and L_3_, respectively), which is also compatible with PDOS analysis.

To better evaluate the nature of transitions associated with NO releasing, additional analysis of the electron/hole (e^–^/h^+^) distribution on CQD_CA_^CYS+TPP^···NO system at the first excited state was conducted. Such an analysis allows us to identify which molecular regions present the most significant changes in charge density after electronic excitation. B3LYP and ωB97X-D functionals were employed for comparison purposes. Figure 7 shows the obtained results, associated with electronic density distributions for the electron and hole (and their overlap) for the first excited state (S_1_) of the CQD_CA_^CYS+TPP^···NO system, considering the distinct exchange-correlation functionals.

**Figure 7:**
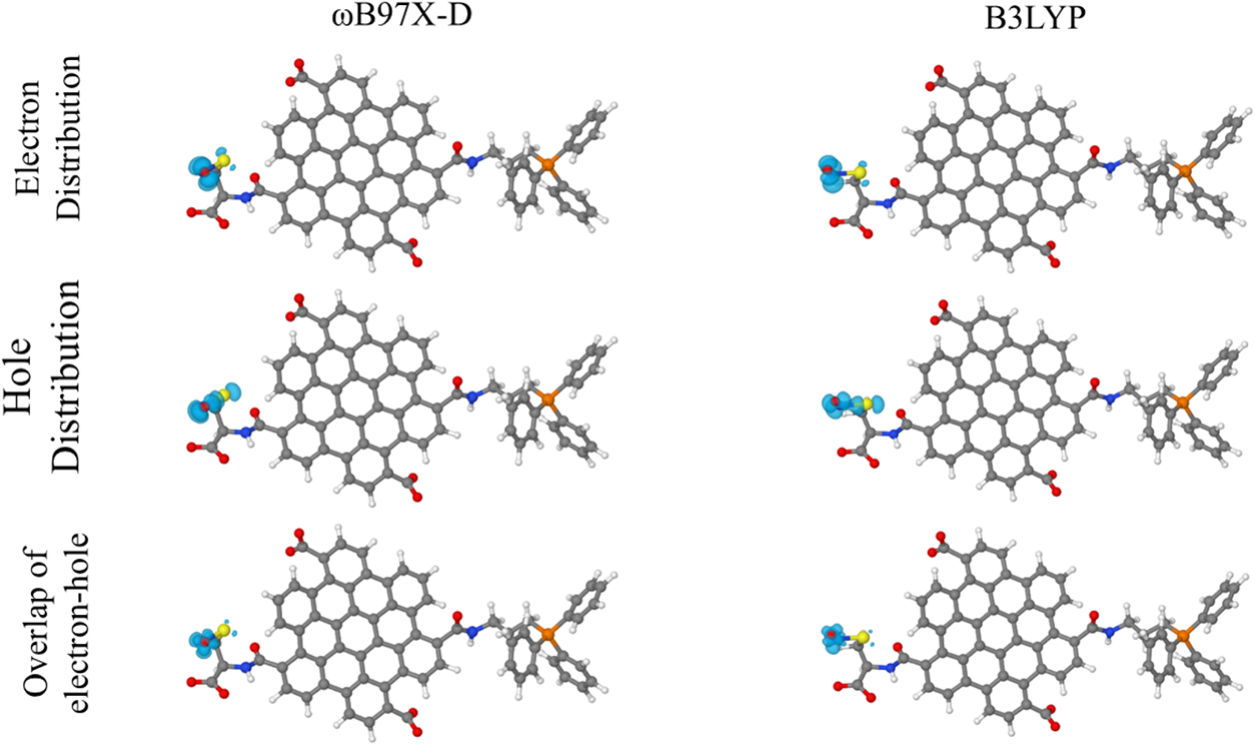
Electron/hole (e^–^/h^+^) density distribution on the CQD_CA_^CYS+TPP^···NO system at the first excited state. Results obtained for DFT/B3LYP/6–31G­(d,p) and DFT/ωB97X-D/6–31G­(d,p) approaches. Both methods show that the e^–^/h^+^ density is mainly localized on the SNO group, consistent with its role in NO release.

Note that the electronic excitation at ∼580 nm is predominantly localized around the S···NO region, with a particular concentration on NO. This result indicates that an effective change in the local electron density around S···NO can be induced by visible light irradiation. A deeper evaluation of the hole and electron distribution reveals a predominant *n* → π* character of this transition (see Figure S7). The antibonding electron distribution suggests that photoexcitation reduces S–N bond density, lowering the release barrier, and promoting NO liberation. This mechanism accounts for the efficient photodynamic response reported for CQD_CA_^CYS+TPP^···NO systems under 550 nm excitation. Similar results are obtained for the B3LYP functional (Figure 7), underscoring the reliability of both approaches in capturing the key electronic features of the system.

To better understand the nature of the 580 nm excitation, transition density matrix (TDM) analysis was conducted for the CQD_CA_^CYS+TPP^···NO system (Figure 8), considering ωB97X-D results. Figure 8A shows a global view of the TDM heat map, evidencing electronic transitions across the whole system. Figure 8B shows the TDM decomposition into selected fragments: 1 = NO, 2 = CYS, 3 = TPP, and 4 = CQD_CA_, to identify specific donor–acceptor interactions and quantify the charge-transfer pathways between these regions.

**Figure 8:**
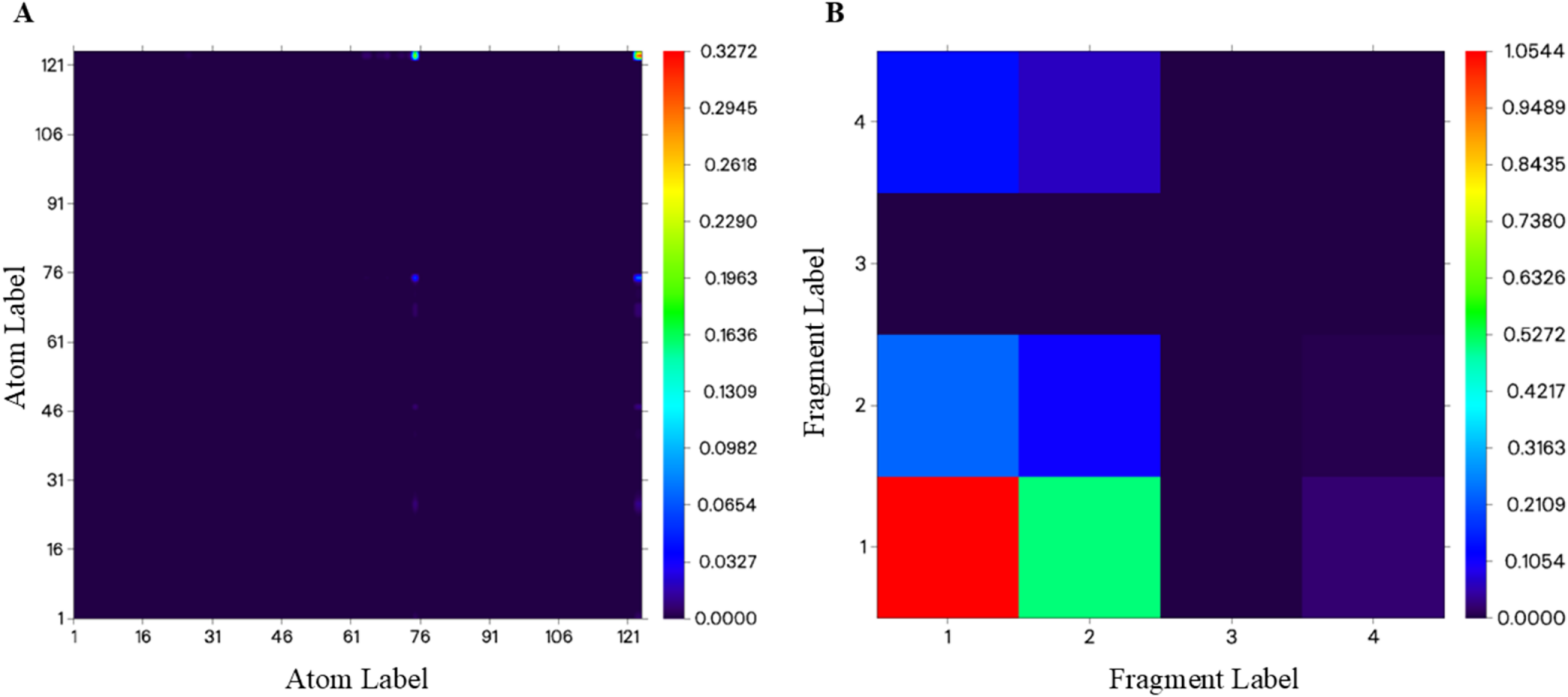
TDM heat-maps of the CQD_CA_^CYS+TPP^···NO system for S_0_ → S_1_ excitation: (A) Overall distribution of electronic transitions across the molecule and (B) fragment-resolved results for 1 = NO, 2 = CYS, 3 = TPP, and 4 = CQD_CA_.

Figure 8A shows the existence of charge-transfer processes between atoms 75 (S from CYS), 123 (N from NO), and 124 (O from NO), with negligible effects in the remaining regions of the system. Figure 8B provides a more detailed view of these photoactivated charge-transfer pathways. The largest contribution to the excitation arises from electronic reorganization within the NO fragment itself, consistent with the primary localization of the h^+^/e^–^ distribution on the S···NO region (Figure 7). A secondary charge transfer from CYS to NO is also observed, highlighting the role of S atom in charge transfer. TPP fragment shows negligible charge-transfer processes, preserving its polarized character, which is essential for electrostatic interactions. Tables 2 and 3 quantitatively confirm the above-described behaviors, showing the contributions of fragments and atoms to the overall photoexcited charge transfer. The data shows that during the S_0_ → S_1_ excitation, CYS fragment donates 0.254 electrons to NO and 0.002 to CQD_CA_ while it receives 0.096 electrons from NO and 0.001 from CQD_CA_, presenting a net charge around +0.159, i.e., it loses 0.159 electrons during the transition. TPP and CQD_CA_ fragments exhibit negligible contributions. The diagonal terms correspond to the amount of intrafragment (atomic) electron redistribution. Table 3 illustrates the atomic contributions by considering only the S–NO system. An effective S → N photoinduced electron transfer process is observed, reinforcing the mechanistic understanding of NO release and the photodynamic response highlighted by the fragment-resolved TDM.

**Table 2:** Interfragment (Donor-to-Acceptor) Charge Transfer (CT) Occurring during Electron Excitation (∼580 nm) in the CQD_CA_^CYS+TPP^···NO System (DFT/ωB97X-D/6-31G­(d,p) Approach) Considering Four Fragments

	acceptor fragment				
donor fragment	NO	CYS	TPP	CQD_CA_	net charge
NO	0.593	0.096	0.000	0.004	–0.164
CYS	0.254	0.040	0.000	0.002	+0.159
TPP	0.000	0.000	0.000	0.000	0.000
CQD_CA_	0.009	0.001	0.000	<0.001	+0.005

**Table 3:** Interatomic (Donor-to-Acceptor) Charge Transfer Occurring during Electron Excitation (∼580 nm) in the CQD_CA_^CYS+TPP^···NO System (DFT/ωB97X-D/6-31G­(d,p) Approach) Centered on SNO Moiety

	acceptor atom			
donor atom	S	N	O	net charge
S	0.084	0.130	0.029	+0.132
N	0.105	0.163	0.037	–0.185
O	0.127	0.197	0.044	+0.053

Given that tumor microenvironments and body mechanics can induce local variations in physiological pH,^41^ additional studies were carried out on CQD_CA_^CYS+TPP^···NO at different protonation states to evaluate their influence on NO release. Figure 9 presents the CAFIs for three CQD_CA_^CYS+TPP^···NO systems, where successive protonation was performed. Our previous results highlighted the relevance of system deprotonation for generating the CQD_CA_^CYS+TPP^···NO complex; in this analysis, we evaluate how the local pH could interfere in the releasing process once the adduct had already been formed.

**Figure 9:**
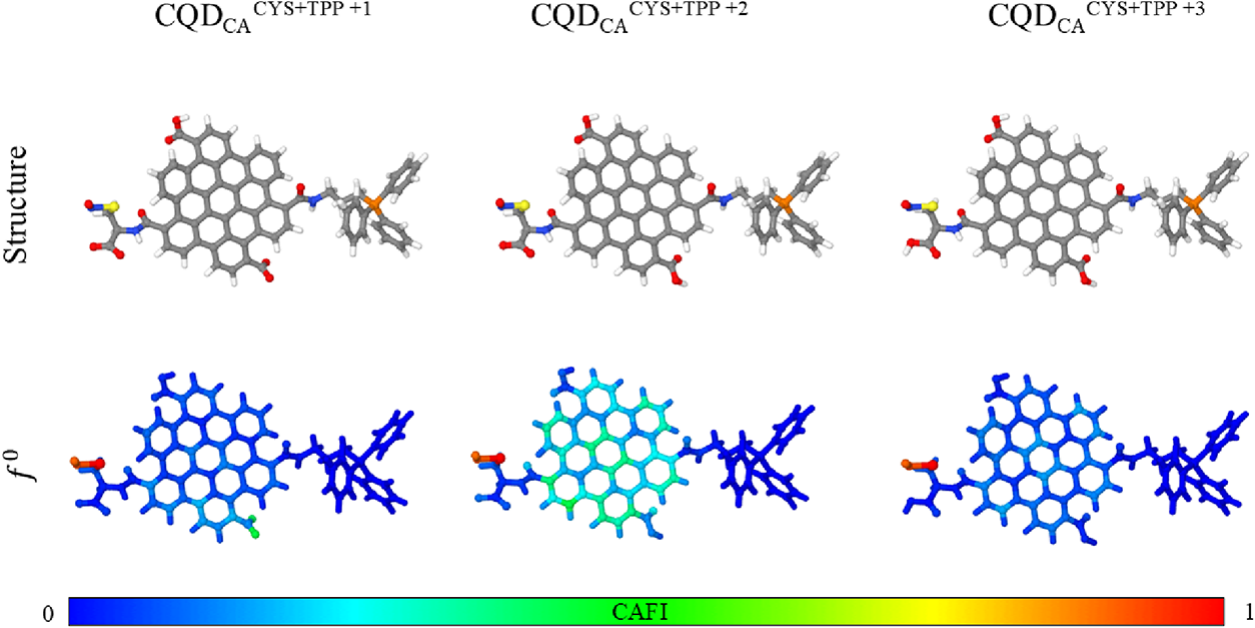
Local reactivity of protonated CQD_CA_^CYS+TPP^···NO. Red and blue sites represent reactive and inert regions in relation to the radicals, respectively. Reactivity remains localized on the SNO group after protonation.

Note that the SNO group remains reactive across all evaluated protonation states, indicating its potential to release NO even under slightly acidic conditions (pH < 6.59 ^17^), a relevant feature given that the local pH in tumor environments typically ranges from 6 to 7.^41^ Compared with the fully deprotonated system (Figure 5), protonated forms showed a modest increase in the reactivity of the CQD_CA_ fragment. Reactivity, assessed via the electrophilic Fukui index (*f* ^–^), revealed that the CQD_CA_^CYS+TPP+2^···NO and CQD_CA_^CYS+TPP+3^···NO systems exhibit slightly enhanced susceptibility to electrophilic attack compared to the fully deprotonated CQD_CA_^CYS+TPP^···NO complex (see Supporting Information for details). This suggests that while NO release remains feasible, protonation may render the system more chemically labile. This trend is reinforced by the analyses of geometric and energetic features of the S···NO bond in our systems, as illustrated in Table 4. Results obtained for isolated NO and simplified HS···NO complex are also presented for comparison purpose.

**Table 4:** Geometrical and Energetic Features of the NO and S···NO-Based Systems

structure	bond type	wiberg bond order	bond length (Å)	S···NO bond angle (°)	system···NO complexation energy (kcal·mol^–1^)
NO	N–O	2.603	1.158		
H–S···NO	S–N	1.165	1.899	114.95	
	N–O	2.386	1.178		
CQD_CA_^CYS+TPP^···NO	S–N	1.229	1.835	115.52	–25.970
	N–O	2.293	1.193		
CQD_CA_^CYS+TPP+1^···NO	S–N	1.228	1.836	115.53	–25.918
	N–O	2.295	1.193		
CQD_CA_^CYS+TPP+2^···NO	S–N	1.226	1.837	115.52	–25.875
	N–O	2.296	1.192		
CQD_CA_^CYS+TPP+3^···NO	S–N	1.184	1.866	115.65	–24.383
	N–O	2.341	1.184		

The complexation energies confirm the formation of stable complexes in all cases. Upon protonation, the S-NO bond length increases (from 1.835 to 1.866 Å), indicating a weakening of this sensitive bond, particularly in the case of CYS residue protonation (CQD_CA_^CYS+TPP+3^···NO). This trend is consistent with the complexation energy analysis for the system···NO. In contrast, the N–O bonds exhibit the opposite behavior relative to the S–N bond.

Such changes could lead to premature NO release under strongly acidic conditions, which may result in undesired side effects if not properly controlled. These findings reinforce the need for careful experimental evaluation of protonated systems responses, particularly in acidic or pathological environments. Nonetheless, it is important to emphasize that under physiological conditions (pH 7.4), the system is expected to exist predominantly in its deprotonated form, which was the primary focus of our simulations. The effects described above are more likely to manifest under conditions of marked acidosis or in acidic microenvironments, such as those found in solid tumors.

## 4 Conclusions

DFT-based calculations have been conducted for functionalized carbon quantum dots to investigate details of the mechanisms involved in the recently reported NO-releasing processes upon visible irradiation, which is very promising for delivery systems.

The results demonstrate that high reactivity on cysteine anchoring centers is obtained only at an appropriate pH, highlighting the relevance of this parameter for the production of CDQ-based active adducts.

TD-DFT-based results evidence the existence of a SNO-localized excited state associated with the experimentally observed NO-releasing process (for excitations around ∼550 nm). This transition displays a predominant *n* → π* character, driving photoinduced S → N electron transfer that weakens the S–NO bond. Altogether, these results provide a coherent mechanistic framework for light-induced NO release, directly linking molecular-level excitations to the observed photodynamic response.

The analysis of protonation suggests that despite maintaining the local reactivity on SNO, the stability of the system may be compromised in very acidic environments, leading to an increased sensitivity to unwanted reactions. However, under typical physiological conditions, the risk of instability is very low.

In summary, our results reveal the complementary roles of the CQD_CA_, CYS, TPP, and NO blocks in stability, photoresponsiveness, and release efficiency in the CQD_CA_^CYS+TPP^···NO system. The COOH groups in CQD_CA_ confer pH-dependent stability, the CYS fragment acts as a protonation-sensitive photodonor, TPP ensures anchoring and surface interactions, and NO serves as the active electronic photoacceptor. This mechanistic understanding provides design principles for engineering CQD-based platforms with enhanced stability, controlled NO release, and reduced off-target effects, paving the way for future optimization and biomedical applications.

## Supplementary Material



## Data Availability

All results in this study are reproducible using the fully optimized structures (for both isolated and adsorbed systems), available at https://drive.google.com/drive/folders/1OIuiusc5dDTzqHfjlzHx7DQVSHGvgOun?usp=drive_link.

## Notes

CAFI results for CQD with zigzag terminations; optical absorption data for the CQD_CA_^CYS+TPP^···NO system using a TD-DFT/B3LYP/6–31G­(d,p) approach; CAFI results (*f* ^–^ and *f* ^*+*^) for the protonated CQD_CA_^CYS+TPP^···NO system; additional tests for CAFI/MEP results (PDF)

## References

[1] Kazemi N., Bakhshandeh B., Dehghani Z., Naghizadeh M. M. (2024). Nanobiomaterials
in Drug Delivery: From Science to Applications. Polym. Bull..

[2] Nair A., Haponiuk J. T., Thomas S., Gopi S. (2020). Natural Carbon-Based
Quantum Dots and Their Applications in Drug Delivery: A Review. Biomed. Pharmacother..

[3] Yang H.-L., Bai L.-F., Geng Z.-R., Chen H., Xu L.-T., Xie Y.-C., Wang D.-J., Gu H.-W., Wang X.-M. (2023). Carbon
Quantum Dots: Preparation, Optical Properties, and Biomedical Applications. Mater. Today Adv..

[4] Das S., Mondal S., Ghosh D. (2024). Carbon Quantum Dots in Bioimaging
and Biomedicines. Front. Bioeng. Biotechnol..

[5] Jin H., Feura E. S., Schoenfisch M. H. (2021). Theranostic
Activity of Nitric Oxide-Releasing
Carbon Quantum Dots. Bioconjugate Chem..

[6] Xu J., Zeng F., Wu H., Hu C., Yu C., Wu S. (2014). Preparation of a Mitochondria-targeted
and NO-Releasing Nanoplatform
and Its Enhanced Pro-Apoptotic Effect on Cancer Cells. Small.

[7] Kulkarni C. A., Fink B. D., Gibbs B. E., Chheda P. R., Wu M., Sivitz W. I., Kerns R. J. (2021). A Novel Triphenylphosphonium Carrier
to Target Mitochondria without Uncoupling Oxidative Phosphorylation. J. Med. Chem..

[8] Nisoli E., Carruba M. O. (2006). Nitric Oxide and
Mitochondrial Biogenesis. J. Cell Sci..

[9] Gao D., Asghar S., Hu R., Chen S., Niu R., Liu J., Chen Z., Xiao Y. (2023). Recent Advances in Diverse Nanosystems
for Nitric Oxide Delivery in Cancer Therapy. Acta Pharm. Sin. B.

[10] Grossi L., Montevecchi P. C. (2002). *S* -Nitrosocysteine and Cystine from
Reaction of Cysteine with Nitrous Acid. A Kinetic Investigation^1^. J. Org. Chem..

[11] Vatanparast M., Shariatinia Z. (2018). Computational
Studies on the Doped Graphene Quantum
Dots as Potential Carriers in Drug Delivery Systems for Isoniazid
Drug. Struct. Chem..

[12] Cui Y., Huang X., Wang T., Jia L., Nie Q., Tan Z., Yu H. (2022). Graphene Quantum Dots/Carbon
Nitride Heterojunction
with Enhanced Visible-Light Driven Photocatalysis of Nitric Oxide:
An Experimental and DFT Study. Carbon.

[13] Yoon H., Park S., Lim M. (2020). Photorelease
Dynamics of Nitric Oxide
from Cysteine-Bound Roussin’s Red Ester. J. Phys. Chem. Lett..

[14] Akhtar M. W., Sunico C. R., Nakamura T., Lipton S. A. (2012). Redox Regulation
of Protein Function via Cysteine S-Nitrosylation and Its Relevance
to Neurodegenerative Diseases. Int. J. Cell
Biol..

[15] Zielonka J., Joseph J., Sikora A., Hardy M., Ouari O., Vasquez-Vivar J., Cheng G., Lopez M., Kalyanaraman B. (2017). Mitochondria-Targeted
Triphenylphosphonium-Based Compounds: Syntheses, Mechanisms of Action,
and Therapeutic and Diagnostic Applications. Chem. Rev..

[16] Alves G. B., Batagin-Neto A. (2023). Local Reactivity on Carbon Quantum
Dots: The Influence
of the Geometries and Chemical Doping for Chemical Sensor Applications. J. Phys. Chem. C.

[17] Orth E. S., Ferreira J. G. L., Fonsaca J. E. S., Blaskievicz S. F., Domingues S. H., Dasgupta A., Terrones M., Zarbin A. J. G. (2016). pKa
Determination of Graphene-like Materials: Validating Chemical Functionalization. J. Colloid Interface Sci..

[18] Dennington, R. ; Keith, T. A. ; Millam, J. M. GaussView Version 6, 2019.

[19] Becke A. D. (1993). Density-Functional
Thermochemistry. III. The Role of Exact Exchange. J. Chem. Phys..

[20] Lee C., Yang W., Parr R. G. (1988). Development
of the Colle-Salvetti
Correlation-Energy Formula into a Functional of the Electron Density. Phys. Rev. B.

[21] Vosko S. H., Wilk L., Nusair M. (1980). Accurate Spin-Dependent Electron
Liquid Correlation Energies for Local Spin Density Calculations: A
Critical Analysis. Can. J. Phys..

[22] Stephens P.
J., Devlin F. J., Chabalowski C. F., Frisch M. J. (1994). Ab Initio Calculation
of Vibrational Absorption and Circular Dichroism Spectra Using Density
Functional Force Fields. J. Phys. Chem. A.

[23] Frisch, M. J. ; Trucks, G. W. ; Schlegel, H. B. ; Scuseria, G. E. ; Robb, M. A. ; Cheeseman, J. R. ; Scalmani, G. ; Barone, V. ; Petersson, G. A. ; Nakatsuji, H. ; Li, X. ; Caricato, M. ; Marenich, A. V. ; Bloino, J. ; Janesko, B. G. ; Gomperts, R. ; Mennucci, B. ; Hratchian, H. P. ; Ortiz, J. V. ; Izmaylov, A. F. ; Sonnenberg, J. L. ; Williams-Young, D. ; Ding, F. ; Lipparini, F. ; Egidi, F. ; Goings, J. ; Peng, B. ; Petrone, A. ; Henderson, T. ; Ranasinghe, D. ; Zakrzewski, V. G. ; Gao, J. ; Rega, N. ; Zheng, G. ; Liang, W. ; Hada, M. ; Ehara, M. ; Toyota, K. ; Fukuda, R. ; Hasegawa, J. ; Ishida, M. ; Nakajima, T. ; Honda, Y. ; Kitao, O. ; Nakai, H. ; Vreven, T. ; Throssell, K. ; Montgomery, J. A., Jr. ; Peralta, J. E. ; Ogliaro, F. ; Bearpark, M. J. ; Heyd, J. J. ; Brothers, E. N. ; Kudin, K. N. ; Staroverov, V. N. ; Keith, T. A. ; Kobayashi, R. ; Normand, J. ; Raghavachari, K. ; Rendell, A. P. ; Burant, J. C. ; Iyengar, S. S. ; Tomasi, J. ; Cossi, M. ; Millam, J. M. ; Klene, M. ; Adamo, C. ; Cammi, R. ; Ochterski, J. W. ; Martin, R. L. ; Morokuma, K. ; Farkas, O. ; Foresman, J. B. ; Fox, D. J. Gaussiañ16 Revision C.01, Gaussiañ; 2016.

[24] Tomasi J., Mennucci B., Cammi R. (2005). Quantum Mechanical
Continuum Solvation
Models. Chem. Rev..

[25] Yang W., Mortier W. J. (1986). The Use of Global
and Local Molecular Parameters for
the Analysis of the Gas-Phase Basicity of Amines. J. Am. Chem. Soc..

[26] Lee C., Yang W., Parr R. G. (1988). Local Softness and Chemical Reactivity
in the Molecules CO, SCN– and H2CO. J.
Mol. Struct.: THEOCHEM.

[27] De
Proft F., Van Alsenoy C., Peeters A., Langenaeker W., Geerlings P. (2002). Atomic Charges, Dipole Moments, and Fukui Functions
Using the Hirshfeld Partitioning of the Electron Density. J. Comput. Chem..

[28] Roy R. K., Pal S., Hirao K. (1999). On Non-Negativity
of Fukui Function Indices. J. Chem. Phys..

[29] Breneman C.
M., Wiberg K. B. (1990). Determining
Atom-centered Monopoles from Molecular
Electrostatic Potentials. The Need for High Sampling Density in Formamide
Conformational Analysis. J. Comput. Chem..

[30] Chai J.-D., Head-Gordon M. (2008). Long-Range Corrected Hybrid Density Functionals with
Damped Atom–Atom Dispersion Corrections. Phys. Chem. Chem. Phys..

[31] Chai J.-D., Head-Gordon M. (2008). Systematic
Optimization of Long-Range Corrected Hybrid
Density Functionals. J. Chem. Phys..

[32] Tortorella S., Talamo M. M., Cardone A., Pastore M., De Angelis F. (2016). Benchmarking
DFT and Semi-Empirical Methods for a Reliable and Cost-Efficient Computational
Screening of Benzofulvene Derivatives as Donor Materials for Small-Molecule
Organic Solar Cells. J. Phys.: Condens. Matter.

[33] Casanova D. (2015). Theoretical
Investigations of the Perylene Electronic Structure: Monomer, Dimers,
and Excimers. Int. J. Quantum Chem..

[34] Modeling of Molecular Properties; Comba, P. , Ed.; Wiley-VCH: Weinheim, 2011.

[35] Lu T., Chen F. (2012). Multiwfn: A Multifunctional
Wavefunction Analyzer. J. Comput. Chem..

[36] Lu T. (2024). A Comprehensive
Electron Wavefunction Analysis Toolbox for Chemists, Multiwfn. J. Chem. Phys..

[37] Liu Z., Lu T., Chen Q. (2020). An Sp-Hybridized
All-Carboatomic Ring, Cyclo[18]­Carbon:
Electronic Structure, Electronic Spectrum, and Optical Nonlinearity. Carbon.

[38] Singh R. J., Hogg N., Joseph J., Kalyanaraman B. (1996). Mechanism
of Nitric Oxide Release from S-Nitrosothiols. J. Biol. Chem..

[39] Jacquemin D., Perpète E. A. (2006). The N→π*
Transition in Nitroso Compounds:
A TD-DFT Study. Chem. Phys. Lett..

[40] Jeong S. Y., Kim M., Kim K., Kim H., Kim Y., Seo J., Lee E., Son K. (2024). The Use of Carbodicarbene for Bio-Based Polyester Synthesis
via Ring-Opening Copolymerization of Cyclic Anhydrides and Epoxides. Eur. Polym. J..

[41] Zhang X., Lin Y., Gillies R. J. (2010). Tumor pH
and Its Measurement. J. Nucl. Med..

