# Supplementary material for: Mechanistic Insights into NO Releasing by Functionalized Carbon Quantum Dots: A DFT Study
Source: ACS Omega. 2025 Oct 31;10(44):52763–72. doi: 10.1021/acsomega.5c06567 (PMC12612914; doi:10.1021/acsomega.5c06567)
Supplement: Supplementary file 1 [file ao5c06567_si_001.pdf]

Supporting Information

Mechanistic insights into NO releasing by functionalized carbon quantum dots: A DFT study

Henrique Rodrigues Souza-Silva [1], Orisson Ponce Gomes [1],João Pedro Dionizio [1], Didier Bégué [2], Paulo Noronha Lisboa-Filho [3,*], Augusto Batagin-Neto [4]

[1] São Paulo State University (UNESP), Graduate Program in Materials Science and Technology (POSMAT), Brazil

[2] Université de Pau et des Pays de l'Adour, E2S UPPA, CNRS, Institut des Sciences Analytiques et Physico-chimie pour l'environnement et les matériaux (IPREM), 64000 Pau, France

[3] São Paulo State University (UNESP), School of Sciences, Bauru/SP 17033-360, Brazil

[4] São Paulo State University (UNESP), Institute of Sciences and Engineering, Itapeva/SP 18409-010, Brazil

* e-mail: paulo.lisboa@unesp.br

S1. CAFI for CQD with zigzag termination (CQD-A)

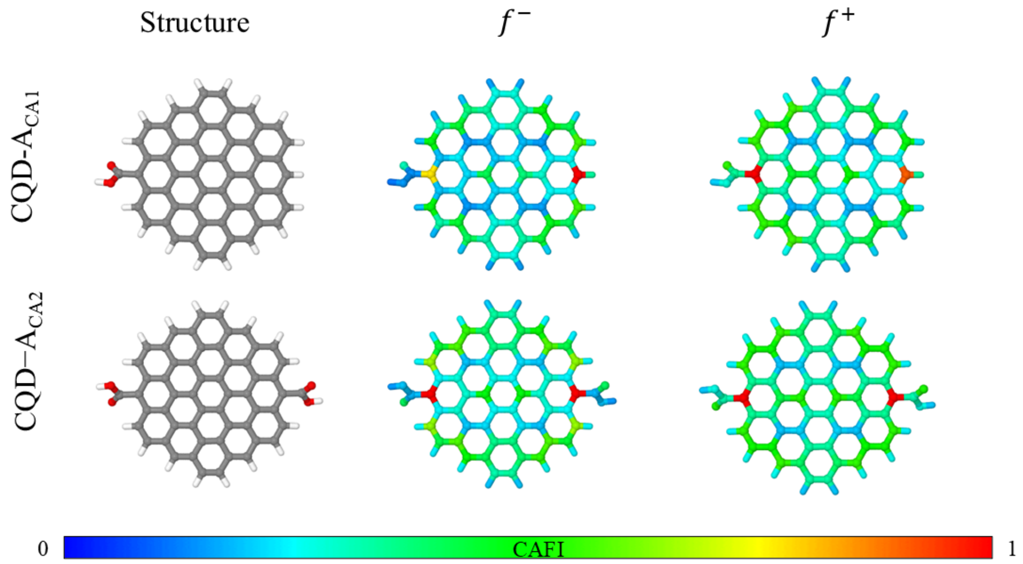

Figure S1. Local reactivity of CQD$_{CAn}$ systems (for n = 1 - 2). Red and blue sites represent reactive and inert regions in relation to electrophiles (f^-) and nucleophiles (f^+), respectively.

S2. CAFI (f^- and f^+) for protonated $CQD_{CA}^{CYS+TPP\cdots}NO$

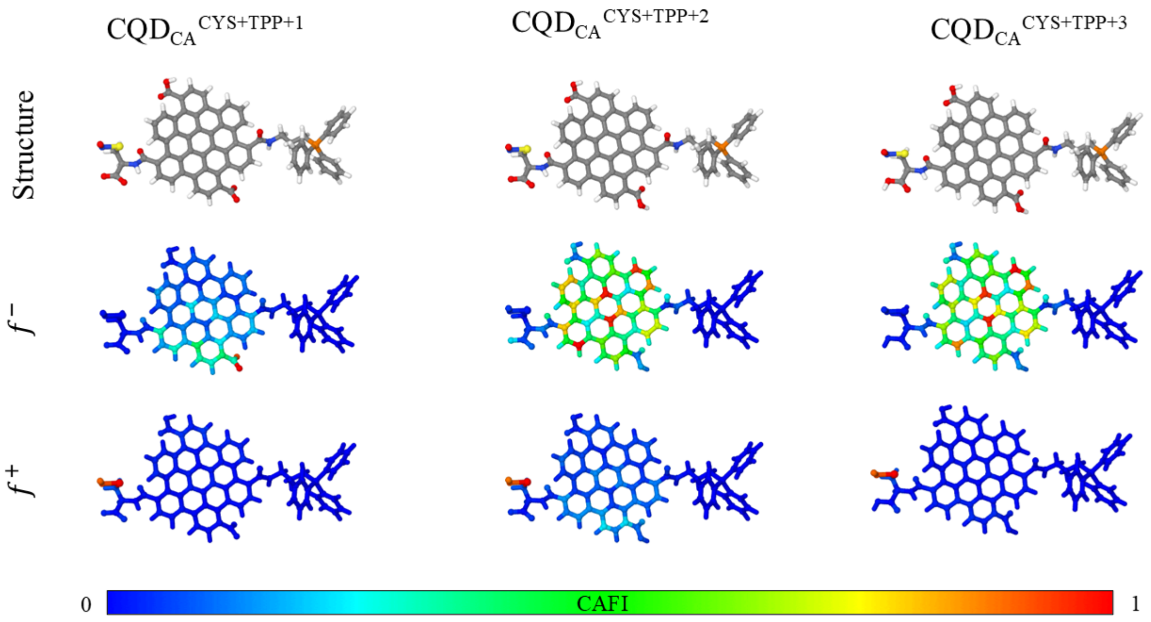

Figure S2. Local reactivity of protonated $CQD_{CA}^{CYS+TPP\cdots}NO$. Red and blue sites represent reactive and inert regions in relation to radicals, respectively.

S3. Influence of the solvent and charge partition method on CAFI

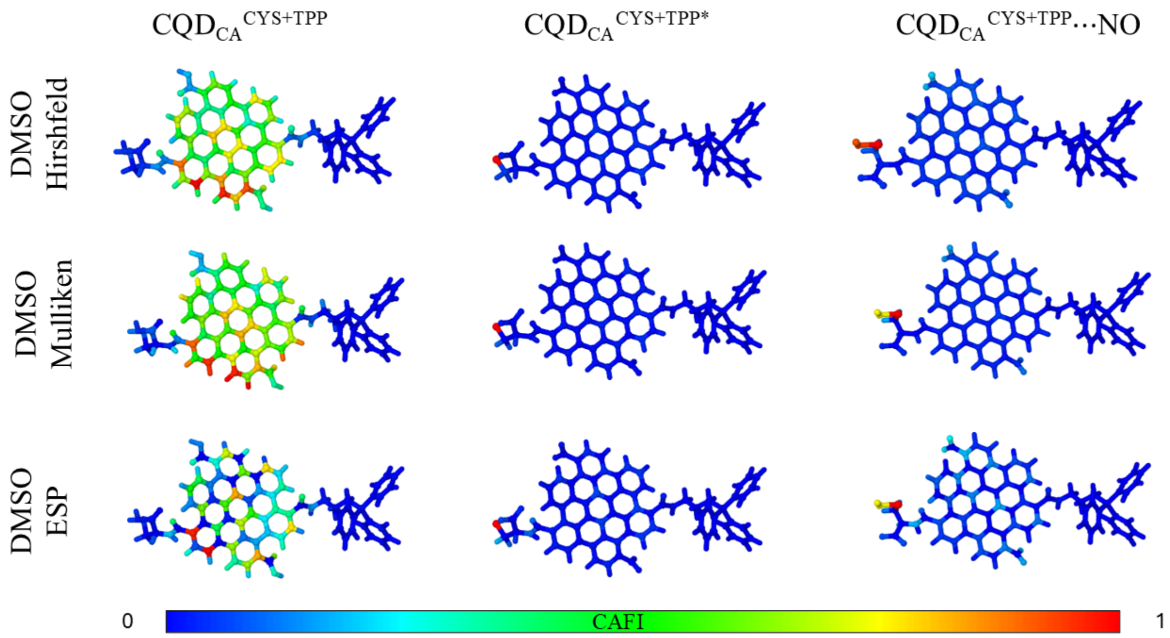

Figure S3. Local reactivity of protonated (#) and deprotonated (*) $CQD_{CA}^{CYS+TPP}$ systems and adsorbed $CQD_{CA}^{CYS+TPP\cdots}NO$ for distinct charge partition method (Hirshfeld, Mulliken and electrostatic derived) and solvent (DMSO instead of water). Red and blue sites represent reactive and inert regions in relation to radicals, respectively.

S4. Influence of solvent on MEP

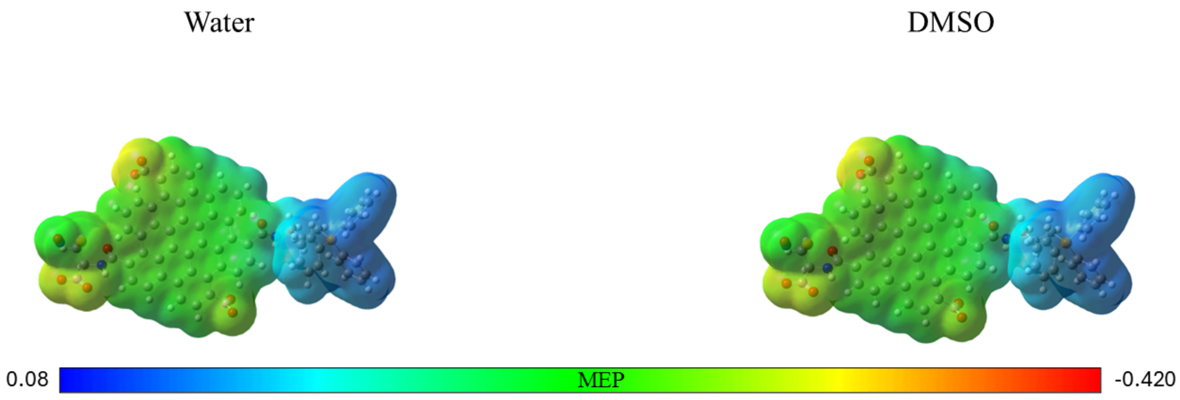

Figure S4. Electrostatic potential (MEP) maps of the $CQD_{CA}^{CYS+TPP\cdots}NO$ system in vacuum, water, and DMSO. The distribution of positive (blue) and negative (red) regions highlights the effect of the solvent environment on the charge localization and surface reactivity of the system.

S5. Influence of grid density on MEP

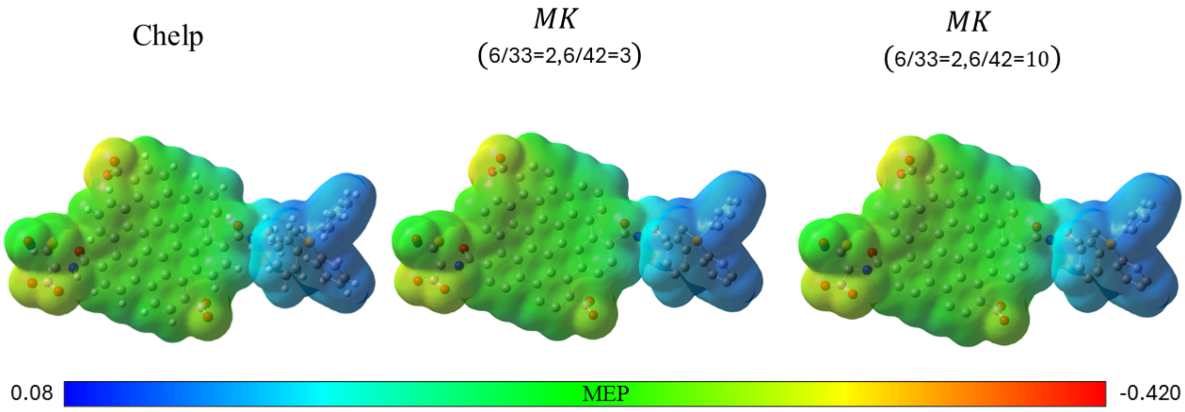

Figure S5. Influence of grid density on MEP: Electrostatic potential (MEP) maps of the $CQD_{CA}^{CYS+TPP\cdots}NO$ system obtained with different grid densities. No significant variation was observed, indicating that the qualitative features of charge localization and reactivity are preserved independently of the grid resolution.

S6. Optical absorption: $CQD_{CA}^{CYS+TPP\cdots}NO$ system for DFT/B3LYP/6-31G(d,p)

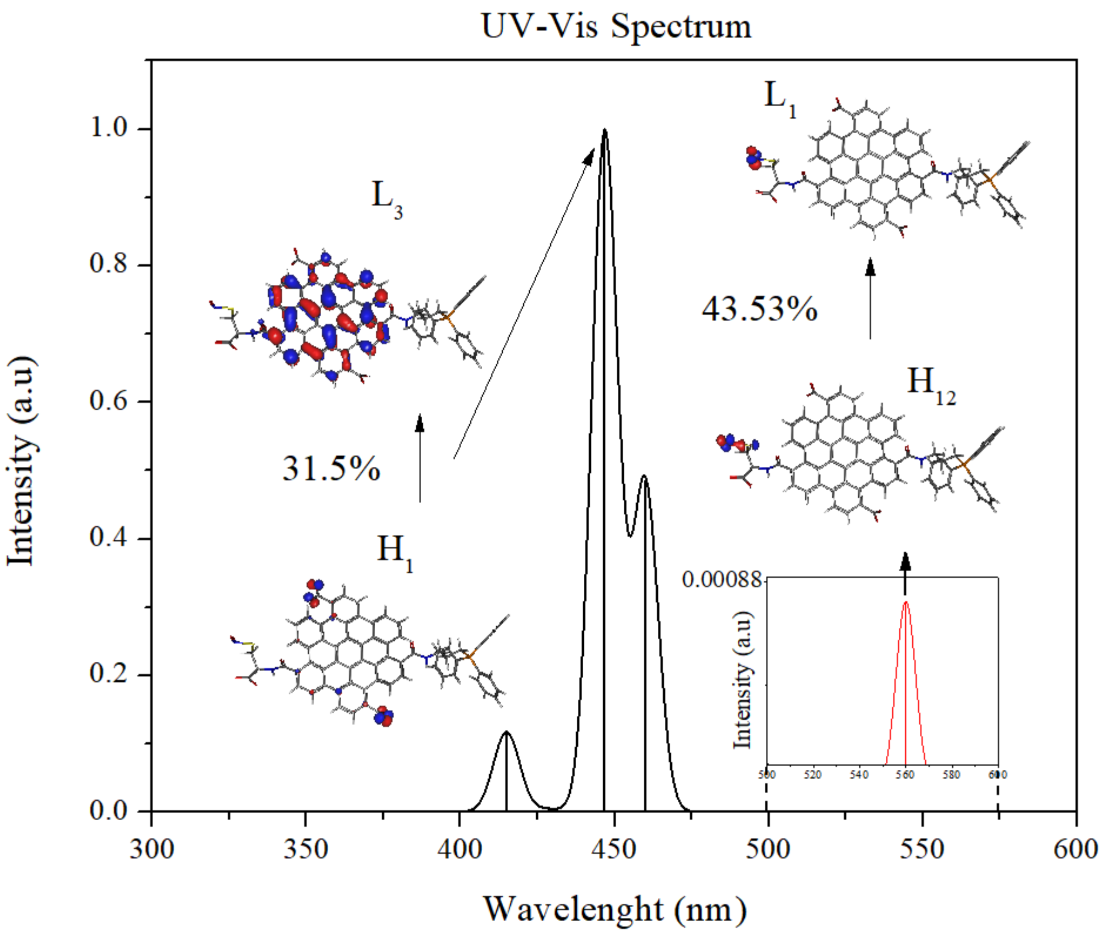

Figure S6. UV-Vis spectrum of $CQD_{CA}^{CYS+TPP\cdots}NO$ systems for DFT/B3LYP/6-31G(d,p)

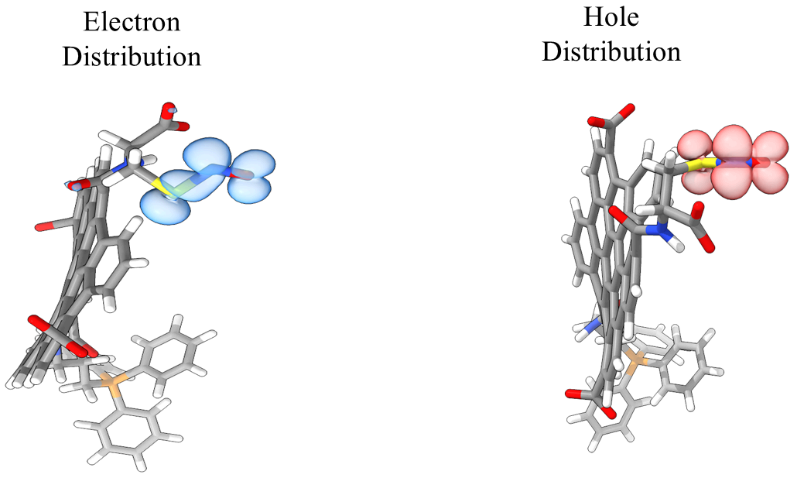

Figure S7. Electron/hole (e^-/h^+) density distribution on $CQD_{CA}^{CYS+TPP\cdots}NO$ system at the first excited state.

Table S1: The first ten excited states and electronic transitions for $CQD_{CA}^{CYS+TPP\cdots}NO$, using B3LYP exchange-correlation functional

Excited State	Energy (eV)	λ_{max} (nm)	*f*	Transitions	c^2
1	2.2141	559.97	0.0003	$H_{18} \rightarrow L_1$<br>$H_{12} \rightarrow L_1$<br>$H_{11} \rightarrow L_1$	0.0108<br>0.4353<br>0.0307
2	2.2908	541.24	0.0000	$H_1 \rightarrow L_1$	0.4933
3	2.5081	494.33	0.0001	$H_5 \rightarrow L_1$<br>$H_4 \rightarrow L_1$<br>$H_3 \rightarrow L_1$<br>$H_2 \rightarrow L_1$	0.0251<br>0.0378<br>0.0615<br>0.3619
4	2.5663	483.12	0.0006	$H_6 \rightarrow L_1$<br>$H_5 \rightarrow L_1$<br>$H_4 \rightarrow L_1$<br>$H_2 \rightarrow L_1$<br>$H_1 \rightarrow L_1$	0.0817<br>0.1649<br>0.1648<br>0.0173<br>0.0532
5	2.6166	473.84	0.0000	$H_2 \rightarrow L_1$<br>$H_1 \rightarrow L_1$	0.4177<br>0.0777
6	2.6960	459.88	0.1818	$H_2 \rightarrow L_2$<br>$H_2 \rightarrow L_3$<br>$H_1 \rightarrow L_2$<br>$H_1 \rightarrow L_3$	0.0306<br>0.0344<br>0.3074<br>0.1033
7	2.7750	446.79	0.3743	$H_2 \rightarrow L_2$<br>$H_1 \rightarrow L_2$<br>$H_1 \rightarrow L_3$	0.0312<br>0.1339<br>0.3155
8	2.8923	428.67	0.0017	$H_8 \rightarrow L_1$<br>$H_7 \rightarrow L_1$	0.4738<br>0.0116
9	2.9371	422.14	0.0000	$H_3 \rightarrow L_1$	0.4978
10	2.9880	414.94	0.0440	$H_1 \rightarrow L_2$<br>$H_1 \rightarrow L_3$<br>$H_1 \rightarrow L_5$	0.3421<br>0.0195<br>0.1094

List of Abbreviations

CAFI, Condensed-to-atoms Fukui indices

CQD, Carbon quantum dot

CQD-A, Carbon quantum dot with zigzag termination

CQD_{CA}, COOH-modified CQD

$CQD_{CA}^{CYS+TPP}$, CYS/TPP-modified CQD_{CA}

$CQD_{CA}^{CYS+TPP\cdots}NO$, $CQD_{CA}^{CYS+TPP}$ complexed with NO

CYS, Cysteine

DFT, Density functional theory

DMSO, Dimethyl sulfoxid

HOMO, Highest occupied molecular orbital

LUMO, Lowest unoccupied molecular orbital

MEP, Molecular electrostatic potential

NO, Nitric oxide

PCM, Polarizable continuum model

PDOS, Partial density of states

R–SNOs, S-nitrosothiols

S_n, Excited state n

TDM, Transition density matrix

TD-DFT, Time-dependent density functional theory

TDOS, Total density of states

TPP, Triphenylphosphonium
